# Coordinated expansion of memory T follicular helper and B cells mediates spontaneous clearance of HCV reinfection

**DOI:** 10.3389/fimmu.2024.1403769

**Published:** 2024-06-10

**Authors:** Mohamed Eisa, Elsa Gomez-Escobar, Nathalie Bédard, Nourtan F. Abdeltawab, Nicol Flores, Sabrina Mazouz, Alizée Fieffé-Bédard, Patrick Sakayan, John Gridley, Mohamed S. Abdel-Hakeem, Julie Bruneau, Arash Grakoui, Naglaa H. Shoukry

**Affiliations:** ^1^ Centre de Recherche du Centre Hospitalier de l’Université de Montréal (CRCHUM), Montréal, QC, Canada; ^2^ Département de microbiologie, infectiologie et immunologie, Université de Montréal, Montréal, QC, Canada; ^3^ Department of Microbiology and Immunology, Faculty of Pharmacy, Cairo University, Cairo, Egypt; ^4^ School of Pharmacy, Newgiza University, Giza, Egypt; ^5^ Department of Medicine, Emory University, Atlanta, GA, United States; ^6^ Département de Médecine familiale et département d’urgence, Université de Montréal, Montréal, QC, Canada; ^7^ Département de Médecine, Université de Montréal, Montréal, QC, Canada

**Keywords:** hepatitis C virus, B cells, neutralizing antibodies, Tfh and immunity, reinfection

## Abstract

**Introduction:**

Follicular helper T cells are essential for helping in the maturation of B cells and the production of neutralizing antibodies (NAbs) during primary viral infections. However, their role during recall responses is unclear. Here, we used hepatitis C virus (HCV) reinfection in humans as a model to study the recall collaborative interaction between circulating CD4 T follicular helper cells (cTfh) and memory B cells (MBCs) leading to the generation of NAbs.

**Methods:**

We evaluated this interaction longitudinally in subjects who have spontaneously resolved primary HCV infection during a subsequent reinfection episode that resulted in either another spontaneous resolution (SR/SR, *n* = 14) or chronic infection (SR/CI, *n* = 8).

**Results:**

Both groups exhibited virus-specific memory T cells that expanded upon reinfection. However, early expansion of activated cTfh (CD4^+^CXCR5^+^PD-1^+^ICOS^+^FoxP3^−^) occurred in SR/SR only. The frequency of activated cTfh negatively correlated with time post-infection. Concomitantly, NAbs and HCV-specific MBCs (CD19^+^CD27^+^IgM^−^E2-Tet^+^) peaked during the early acute phase in SR/SR but not in SR/CI. Finally, the frequency of the activated cTfh1 (CXCR3^+^CCR6^−^) subset correlated with the neutralization breadth and potency of NAbs.

**Conclusion:**

These results underscore a key role for early activation of cTfh1 cells in helping antigen-specific B cells to produce NAbs that mediate the clearance of HCV reinfection.

## Introduction

T follicular helper (Tfh) cells play a crucial role in helping B cells and in the formation of germinal centers, affinity maturation, and development of plasma cells and memory B cells (MBCs) during primary viral infections and vaccinations (1, 2). However, their role during recall responses and their correlation with the production of neutralizing antibodies upon reinfection are understudied (3). Hepatitis C virus (HCV) infection represents an ideal model to study this question with two dichotomous outcomes where approximately 30% of acutely infected individuals resolve spontaneously while the rest develop chronic infection. Despite the resolution of primary HCV infection, people who inject drugs (PWID) remain at high risk of HCV exposure and reinfection (4–8), thus representing a natural experimental rechallenge framework to study memory immune responses against human viral infection. Spontaneous resolution of primary HCV is associated with both T-cell and antibody responses. However, although the resolution of primary infection leads to the generation of long-lived memory T cells (9, 10), antibody responses proceed to decline rapidly (10–12). Virus-specific circulating T follicular helper (cTfh) CD4^+^ T cells expand during acute HCV (13) and the frequencies of CXCR3^+^ cTfh (cTfh1) positively correlate with the magnitude and breadth of HCV-neutralizing antibody responses (14). We have demonstrated that early expansion of activated cTfh1 expressing interleukin 21 (IL-21), CD40L, and interferon-γ (IFN-γ) is associated with the expansion of HCV-specific MBCs in spontaneous resolvers of acute HCV infection (11).

Early expansion of HCV-specific MBCs and the production of neutralizing antibodies (NAbs) are associated with clearance of acute primary HCV infection and reinfection (11, 12, 15–21). NAbs with exceptionally high neutralization breadth and potency were identified in HCV elite neutralizers and are associated with the use of the VH1–69 heavy-chain gene segment (22, 23). HCV reinfection and repeated exposure to viruses with antigenically related, antibody-sensitive E1E2s glycoproteins lead to the generation of potent broadly neutralizing antibodies (bNAbs) (16). Resolution of HCV reinfection is also associated with expansion of HCV-specific CD4 and CD8 T cells (18, 24). We have demonstrated that spontaneous resolution of HCV reinfection is associated with an early plasma cell transcriptomic signature, variable levels of NAbs, and early expansion of HCV-specific MBCs and CD8 T cells (17), indicating that cooperative effort between NAbs and T cells is required for long-term protection against HCV. How much of this response is driven by memory CD4 T-cell help and Tfh–MBC interaction upon reinfection has not been studied thus far.

Here, we investigated the longitudinal expansion of activated cTfh and HCV-specific MBCs during HCV reinfection. We observed the earlier expansion of activated cTfh and HCV-specific MBCs in resolvers as opposed to chronic subjects. Furthermore, the frequencies of activated cTfh1 were associated with the neutralization breadth and potency of antibodies in resolvers. Our data suggest a cooperative role for activated HCV-specific cTfh cells and NAbs in the clearance of HCV during re-exposure.

## Materials and methods

### Human study participants

Study subjects were recruited among PWID who were participating in the Montreal Hepatitis C cohort (HEPCO, study protocol approval number: SL 05.014). All subjects were HIV-negative. HCV reinfection was defined by an HCV-positive RNA test following two consecutive negative tests >30 days apart (Cobas Ampliprep/Cobas TaqMan HCV Qualitative Test, version 2.0; limit of detection: 15 IU/ml). The median between the last negative and first positive HCV RNA test was used to determine the estimated date of reinfection (EDI). Study subjects were considered spontaneous resolvers of reinfection if HCV RNA was negative at 6 months post-EDI, while chronics were defined by a positive test.

### IFN-γ enzyme-linked immunospot assay

HCV-specific T-cell responses were measured using an IFN-γ enzyme-linked immunospot (ELISpot) assay, as previously described (25) with an input of 2 × 10^5^ PBMCs/well against 11 pools of overlapping peptides spanning the entire HCV polyprotein corresponding to genotype (Gt) 1a (H77), 1b (J4), or 3a (K3a/650) sequences (BEI Resources, Manassas, VA, USA). For some subjects, we used the ELISpot Flex Human IFN-γ (ALP) kit (Mabtech, Cincinnati, OH, USA). All assays were performed directly *ex vivo* on frozen PBMCs. Specific spot-forming cells (SFCs) were calculated as the mean number of spots in test wells minus the mean number of spots in negative control wells and normalized to SFC/10^6^ PBMCs. Early and late acute time points used for this assay differed from the ones mentioned in **Supplementary Table 1** for subjects SR/SR-4, SR/SR-6, and SR/CI-2. Pre-reinfection time points of SR/CI-1 and SR/CI-2 were also different.

### HCV E2 tetramer

Biotinylated E2 monomers were generated by the laboratory of Dr. Arash Grakoui (26) and prepared as previously described (17) by five times addition of 3.75 μl phycoerythrin (PE)-labeled ExtrAvidin (Millipore Sigma, Oakville, ON, Canada) or allophycocyanin (APC)-labeled streptavidin (Invitrogen, Carlsbad, CA, USA) to 4.5 μl of E2 monomers (2.29 mg/ml) followed by 10 min of incubation at room temperature (RT) after each addition. For tetramer staining, 0.56 μl of tetramer-PE and 0.56 μl of tetramer-APC were used in 100 μl of RPMI 10 [RPMI medium (Wisent, St-Jean-Baptiste, QC, Canada) supplemented with 10% fetal bovine serum (FBS) (Corning, Corning, NY, USA) and 0.01% sodium azide (Thermo Fisher Scientific, Waltham, MA, USA)].

### Flow cytometry

Cryopreserved PBMCs were thawed and washed twice with RPMI 10 and then washed twice with FACS buffer (PBS 1×, 1% FBS, 0.01% sodium azide). For the identification of E2-specific MBCs, cells were incubated for 10 min at RT with Human BD Fc Block (BD Biosciences, Franklin Lakes, NJ, USA) and then stained with biotinylated E2 tetramers for 30 min at RT. Cells were then washed twice with FACS buffer and stained for 30 min at 4°C with surface markers (see **Supplementary Table 2**: Panel #1 for antibodies) and viability dye [LIVE/DEAD fixable aqua dead cell stain kit (Thermo Fisher Scientific)] to identify live cells. Following two washes with FACS buffer, cells were fixed using 1% formaldehyde (Millipore Sigma). For cTfh phenotyping, cells were stained for 30 min at 4°C with surface markers (see **Supplementary Table 2**: Panel #2 for antibodies) and viability dye. Cells were washed again and permeabilized using FoxP3 fixation and permeabilization buffer (Thermo Fisher Scientific) for 20 min at 4°C. Intracellular staining was performed for 30 min at 4°C. Cells were then washed twice with PermWash buffer and fixed using 1% formaldehyde. Multiparameter flow cytometry was performed at the flow cytometry core of the CRCHUM using a BD LSRFortessa instrument equipped with five lasers [UV (355 nm), violet (450 nm), blue (488 nm), yellow-green (561 nm), and red (640 nm)] and the FACSDiva version 9.2 (BD Biosciences). FCS data files were analyzed using FlowJo (version 10.8.1 for Mac; BD Biosciences).

### Activation-induced markers assay

Cryopreserved PBMCs were thawed and then washed twice with RPMI 5 [RPMI medium supplemented with 5% human Serum (Wisent) and 1% penicillin/streptomycin (Wisent)]. Cells (1 to 4 × 10^6^) were then incubated in 1 ml of RPMI 5 for 3 h at 37°C, 5% CO_2_ in 5 ml of polypropylene push cap tubes (Thermo Fisher Scientific). Antibodies for the chemokine receptors CCR4, CCR6, CCR7, CXCR3, and CXCR5 were added to the culture, and the cells were further incubated for 15 min at 37°C, 5% CO_2_, followed by the addition of a CD40 blocking antibody (Miltenyi, Gaithersburg, MD, USA) to prevent the interaction of CD40L with CD40 and its subsequent downregulation. Cells were then stimulated for 18 h at 37°C, 5% CO_2_ with 1 µg/ml of HCV overlapping peptide pools showing the highest response in ELISpot. Cells stimulated with 1 µg/ml of staphylococcal enterotoxin B (SEB) (Toxin Technology Inc., Sarasota, FL, USA) and unstimulated cells served as positive and negative controls, respectively. Cells were then collected and transferred to a 96-well V-bottom plate, washed twice with FACS buffer, and stained for 30 min at 4°C with surface markers (see **Supplementary Table 2**: Panel #3 for antibodies) and viability dye. Following two washes, cells were fixed using 1% formaldehyde. Multiparameter flow cytometry was performed as described above.

### HCV E2 and NS3 ELISA

ELISA was performed as described previously (11) with 0.5 μg/ml in-house generated H77-E2 and J6-E2 (26) or NS3 protein [RayBiotech, Peachtree Corners, GA, USA recombinant NS3 (Gt 1a) amino acids 1192 to 1459]. Plasma samples from the subjects were diluted starting at 1:250 with four-fold dilutions up to 1:16,000. All samples were tested in duplicates. The difference in optical densities (OD450–570) was determined by subtracting the mean absorbance at 570 nm (background) from the mean absorbance at 450 nm. Absorbance measurements were performed on a Synergy 4 Microplate Reader (BioTek Instruments, Winooski, VT, USA).

### Cell lines and cell culture

CD81-knockout (KO) human embryonic kidney 293T (HEK 293T) (kindly provided by Drs. Joe Grove, University of Glasgow and Justin Bailey, Johns Hopkins) and human hepatoma Huh-7 (kindly provided by Dr. Charles Rice, The Rockefeller University) cell lines were cultured in Dulbecco’s modified Eagle’s medium (DMEM) (Thermo Fisher Scientific) supplemented with 10% FBS and maintained at 37°C and 5% CO_2_.

### Generation of HCV pseudoparticles

HCV pseudoparticles (HCVpp) were produced as described previously (27). Briefly, CD81-KO HEK293T cells were seeded at 1 × 10^6^ cells/well on six-well plates. The following day, cells were transfected with pNL4–3.Luc.R-E-plasmid containing the env-defective HIV proviral genome, pAdVantage, and HCV E1E2 plasmids (UKNP1.11.6, 1a154, UKNP4.2.2, 1a72, 1b58, UKNP3.1.2, or UKNP1.18.1) (kindly provided by Dr. Justin Bailey) (28) using Lipofectamine 2000 (Thermo Fisher Scientific) following the manufacturer’s instructions. For mock HCVpp, HCV E1E2 plasmid was not added to the transfection mix. Media were changed 24 h later, and supernatants were collected at 48 and 72 h after transfection. Supernatants were filtered through a 0.45-μm filter, separated into aliquots, and stored at −80°C until future use.

### HCVpp neutralization assays

Neutralization assays were performed as previously described elsewhere (27). Huh-7 cells were plated on wells of sterile 96-well white plates (Thermo Fisher Scientific) at a density of 1.5 × 10^4^ cells/well. The next day, plasma aliquots were incubated at 56°C for 30 min to inactivate the complement and centrifuged at 1,200×*g* for 5 min. Fixed plasma dilutions (1:25) were added to an equal volume of either UKNP1.11.6, 1a154, UKNP4.2.2, 1a72, 1b58, UKNP3.1.2, or UKNP1.18.1 E1E2 HCVpp or mock HCVpp. Dilutions of HCV-negative and HCV-positive (chronically infected donor) plasma were included as negative and positive controls, respectively. Plasma-HCVpp complexes (final plasma dilution 1:50) were incubated at 37°C for 1 h. A volume of 100 µl of plasma-HCVpp mixture was added to cell monolayers in duplicate and incubated for 5 to 6 h at 37°C and 5%CO_2_, after which the mixture was discarded and replaced with phenol red-free DMEM (Wisent) supplemented with 10% FBS and 4 mM of L-glutamine (Wisent). After 48 h, cells were lysed in 50 μl of 1× Luciferase cell culture lysis buffer (Promega, Madison, WI, USA) at RT. Luminescence was detected by adding 50 µl of luciferase reagent (Promega) and measuring relative light units (RLU) on a Synergy 4 Microplate Reader (BioTek Instruments). Percent neutralization was calculated using the following equation: % neutralization = [1 − (RLU_infection time point_ − RLU_mock_)/(RLU_negative_ − RLU_mock_)]. Neutralizing breadth was defined as the number of HCVpp (of the 7 HCVpp used) neutralized with a % neutralization >50%, and neutralizing potency was calculated as the geometric mean of the % neutralization from the assays against 7 HCVpp for a given time point.

### Statistics

Statistical analyses were performed with Prism version 10.1.0 (GraphPad, Boston, MA, USA). Details of the tests (including the number of data points (*n*) and *P*-values) are provided in each figure legend. Differences between groups in longitudinal analyses were determined by two-way repeated measure ANOVA with Tukey’s *post-hoc* test. Comparisons that did not include multiple time points were examined by the two-tailed Mann–Whitney *U* test. Correlations between variables were examined using Spearman’s test. Non-linear regression was used to calculate antibody titers. For all statistical tests, *P*-values less than 0.05 were considered significant.

## Results

### Study design and characteristics of HCV-reinfected subjects

We examined the HCV-specific immune response during documented HCV reinfection episodes after spontaneous resolution in a group of PWID participating in the Montreal Hepatitis C cohort (HEPCO) study as described in the *Materials and methods* (29, 30) (*n* = 22; **Table 1**). Of these reinfection cases, 14 subjects spontaneously resolved the reinfection episode, hereinafter termed resolvers or SR/SR, while eight subjects developed chronic infection, hereinafter termed chronics or SR/CI. The detailed demographics and clinical characteristics of the subjects are summarized in **Supplementary Table 1**. We analyzed the response at key time points during reinfection: pre-reinfection (variable), early acute (<3 months post-EDI), late acute (3–7 months post-EDI), and follow-up (>7 months post-EDI). Most SR/SR (64.2%) and SR/CI (62.5%) were reinfected with HCV Gt 1, while only 1 of 14 SR/SR and 2 of 8 SR/CI were reinfected with HCV Gt 3a (**Table 1**). Both groups had pre-existing virus-specific memory T-cell responses that expanded during reinfection as measured in IFN-γ ELISpot assay against overlapping peptide pools covering the entire HCV polyprotein. During early acute reinfection, both groups had comparable total T-cell responses that were sustained during the late acute time point primarily in SR/SR (**Supplementary Figure 1**).

**Table 1:** Summary of the subjects’ demographics and clinical characteristics.

	Spontaneous resolution of HCV reinfection (SR/SR) *n* = 14	Chronic HCV reinfection (SR/CI) *n* = 8
**Sex (M/F)**	12/2	6/2
**Age at reinfection (years)**	Range: 31–56 Mean: 41.6	Range: 27–56 Mean: 38
% Ethnicity (*n*)		
**Caucasian** **Unknown**	78.6 (11) 21.4 (3)	62.5 (5) 37.5 (3)
% HCV reinfection genotype (*n*)		
**1a** **1b** **1a–1b** **1** **3a** **ND**	21.4 (3) 7.1 (1) 7.1 (1) 28.6 (4) 7.1 (1) 28.6 (4)	50 (4) 12.5 (1) – – 25 (2) 12.5 (1)

ND, not determined.

### Early expansion of activated cTfh cells in resolvers during acute HCV reinfection

**Figure 1:**
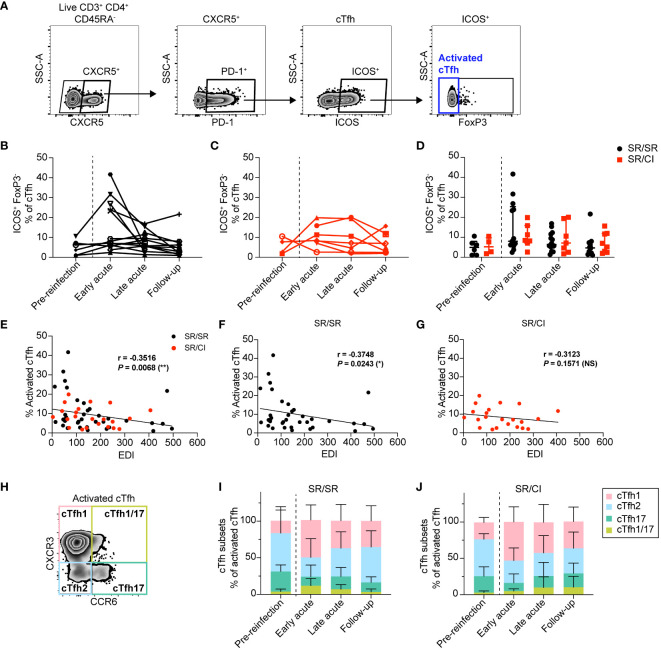
Activated circulating CD4 T follicular helper (cTfh) cells expand during early acute reinfection in resolvers (SR/SR) but not in chronics (SR/CI). **(A)** Representative gating strategy showing activated cTfh cells (CD3^+^CD4^+^CD45RA^−^CXCR5^+^PD1^+^ICOS^+^FoxP3^–^) from the PBMCs of HCV-reinfected subjects. **(B, C)** Longitudinal frequencies of activated cTfh cells at different time points in SR/SR [**(B)** *n* = 14, black] and SR/CI [**(C)** *n* = 8, red]. **(D)** Combined data from **(B–C)**, presenting the median with interquartile range for each group. **(E–G)** Scatter plots for Spearman’s rank correlation between the frequency of activated cTfh cells and estimated date of reinfection (EDI), data combined from SR/SR and SR/CI **(E)**, SR/SR only **(F)**, or SR/CI only **(G)**; the correlation coefficients (*r*-values) and *P*-values are shown. *P < 0.05; **P < 0.01; NS, non-significant. **(H)** Representative gating strategy for different subsets of activated cTfh: cTfh1 (CXCR3^+^CCR6^−^), cTfh2 (CXCR3^−^CCR6^−^), cTfh17 (CXCR3^−^CCR6^+^), and cTfh1/cTfh17 (CXCR3^+^CCR6^+^). **(I, J)** Phenotypic characterization showing the polarization of activated cTfh cells in SR/SR **(I)** and SR/CI **(J)**.

First, we examined longitudinally the expansion of total activated cTfh identified as CD3^+^CD4^+^CD45RA^−^CXCR5^+^PD1^+^ICOS^+^FoxP3^–^ (**Figure 1A**) in SR/SR and SR/CI. We observed higher frequencies of total activated cTfh at the early acute time point in SR/SR (**Figure 1B**) compared to SR/CI subjects (**Figure 1C**), although there was no significant difference between the two groups (**Figure 1D**). In both groups, frequencies of activated cTfh diminished at late acute and follow-up. Notably, at early acute, five SR/SR subjects (SR/SR-6, SR/SR-9, SR/SR-11, SR/SR-12, and SR/SR-14) exhibited high frequencies of activated cTfh ranging from 23.4% to 41.7% (**Figure 1B**), whereas in the SR/CI group, activated cTfh did not surpass 19.9% (**Figure 1C**). Given that the time points analyzed are based on EDI, we reasoned that time points may not be all synchronized and that transient expansion of cTfh might have been missed in some subjects. Hence, we examined the correlation between the frequency of activated cTfh and the EDI. Indeed, there was an overall significant negative correlation between these two parameters in all subjects (*r* = −0.3516, *P* = 0.0068) (**Figure 1E**), and when stratified into the two groups, this correlation remained significant only in SR/SR (*r* = −0.3748, *P* = 0.0243) (**Figure 1F**) but not in SR/CI (*r* = −0.3123, *P* = 0.1571) (**Figure 1G**), underscoring the transient nature of this expansion in resolvers and the effect of time after reinfection. We then examined the distribution of distinct subsets of activated cTfh based on the expression of the chemokine markers CXCR3 and CCR6 (31–35) (**Figure 1H**). In both groups, the activated cTfh were predominantly cTfh2 (CXCR3^−^CCR6^−^) (median: SR/SR = 52.39%, SR/CI = 50.82%) prior to reinfection but became predominantly cTfh1 (CXCR3^+^CCR6^−^) (median: SR/SR = 60.34%, SR/CI = 44.89%) at early acute followed by cTfh2 (median: SR/SR = 17.5%, SR/CI = 31.25%), with smaller subsets demonstrating cTfh17 (CXCR3^−^CCR6^+^) (median: SR/SR = 5.6%, SR/CI = 7.19%) and cTfh1/17 (CXCR3^+^CCR6^+^) (median: SR/SR = 12.62%, SR/CI = 4.73%) phenotypes (**Figures 1I, J**). In summary, resolvers exhibited transient expansion of activated cTfh1 cells at the early acute time point, potentially contributing to clearance of HCV reinfection.

### Resolvers' early cTfh are HCV-specific

**Figure 2:**
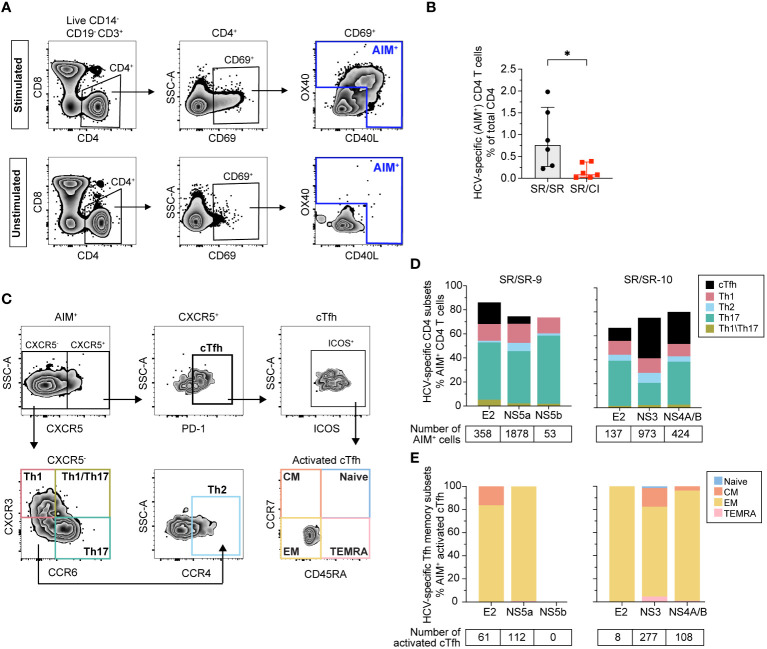
Higher frequencies of AIM^+^ CD4^+^ T cells including cTfh in resolvers at early acute reinfection. **(A)** Representative gating strategy of HCV-specific (AIM^+^) CD4^+^ T cells (CD14^−^CD19^−^CD3^+^CD8^−^CD4^+^CD69^+^) expressing CD40L and/or OX40. Results shown are from SR/SR-9 which were either stimulated with the HCV peptide pool NS5a (top) or unstimulated (bottom). **(B)** Frequencies of AIM^+^ CD4^+^ T cells in SR/SR-9 and SR/SR-10 (black dots) versus SR/CI-3 and SR/SR-5 (red squares). Data are shown as median with interquartile range for each group of subjects. Two-tailed Mann–Whitney test. *P < 0.05. **(C)** Representative gating strategy for phenotypic characterization of AIM^+^ CD4^+^ T-cell subsets from SR/SR as cTfh (CXCR5^+^PD-1^+^), Th1 (CXCR5^−^CXCR3^+^CCR6^−^), Th2 (CXCR5^−^CXCR3^−^CCR6^−^CCR4^+^), Th17 (CXCR5^−^CXCR3^−^CCR6^+^), and Th1/Th17 (CXCR5^−^CXCR3^+^CCR6^+^). Also shown is the representative gating for different memory subsets among AIM^+^-activated (ICOS^+^) cTfh cells: naive (CCR7^+^CD45RA^+^), central memory (CM) (CCR7^+^CD45RA^−^), effector memory (EM) (CCR7^−^CD45RA^−^), and terminally differentiated effector memory T cells expressing CD45RA (TEMRA) (CCR7^−^CD45RA^+^). **(D)** Stacked bar charts showing phenotypic characterization of AIM^+^ CD4^+^ T cells in SR/SR and their polarization toward cTfh, Th1, Th2, Th17, and Th1/Th17 in response to stimulation with the indicated peptide pools (*x*-axis), presented as percentage of total AIM^+^ CD4 T cells. The number of AIM^+^ events gated from each peptide pool is indicated below the graphs. **(E)** Stacked bar charts showing the frequencies of different memory subsets as a percentage of AIM^+^-activated cTfh cells from SR/SR in response to stimulation with the indicated peptide pools (*x*-axis). The number of activated cTfh gated from each peptide pool is indicated below the graphs.

To confirm that the expanded cTfh are virus-specific, we performed an activation-induced markers (AIM) assay at early acute in two SR/SR (SR/SR-9 and SR/SR-10) and two SR/CI subjects (SR/CI-3 and SR/CI-5). We used the peptide pools that gave the highest response in the IFN-γ ELISpot assay, in addition to E2 as it is the main target of NAbs that require Tfh help. HCV-specific (AIM^+^) CD4 T cells were identified as CD14^−^CD19^−^CD3^+^CD4^+^CD8^−^CD69^+^ T cells expressing the activation markers CD40L and/or OX40. The representative gating strategy is presented in **Figure 2A**. SR/SR subjects exhibited significantly higher frequencies of HCV-specific CD4 T cells (*P* = 0.026) than SR/CI subjects (**Figure 2B**).

Next, we examined the distribution of different CD4 subsets among AIM^+^ T cells. The representative gating strategy is presented in **Figure 2C**. HCV-specific cells in SR/SR were predominantly Th17 (CXCR5^−^CXCR3^−^CCR6^+^), followed by Th1 (CXCR5^−^CXCR3^+^CCR6^−^) and Tfh (CXCR5^+^PD-1^+^), while Th2 (CXCR5^−^CXCR3^−^CCR6^−^CCR4^+^) and Th1/17 (CXCR5^−^CXCR3^+^CCR6^+^) represented a small percentage of AIM^+^ cells (**Figure 2D**). Moreover, HCV-specific cTfh cells in the two SR/SR subjects had an effector memory phenotype (CD45RA^−^CCR7^–^) (**Figure 2E**). The frequency of HCV-specific CD4 T cells in SR/CI was too low to accurately phenotype the cells. In summary, SR/SR showed significantly higher frequencies of effector memory HCV-specific CD4 T cells compared to SR/CI during early acute reinfection. These CD4 T cells were enriched in Th17, Th1, and cTfh phenotypes.

### Early expansion of HCV-specific MBCs in resolvers during reinfection

**Figure 3:**
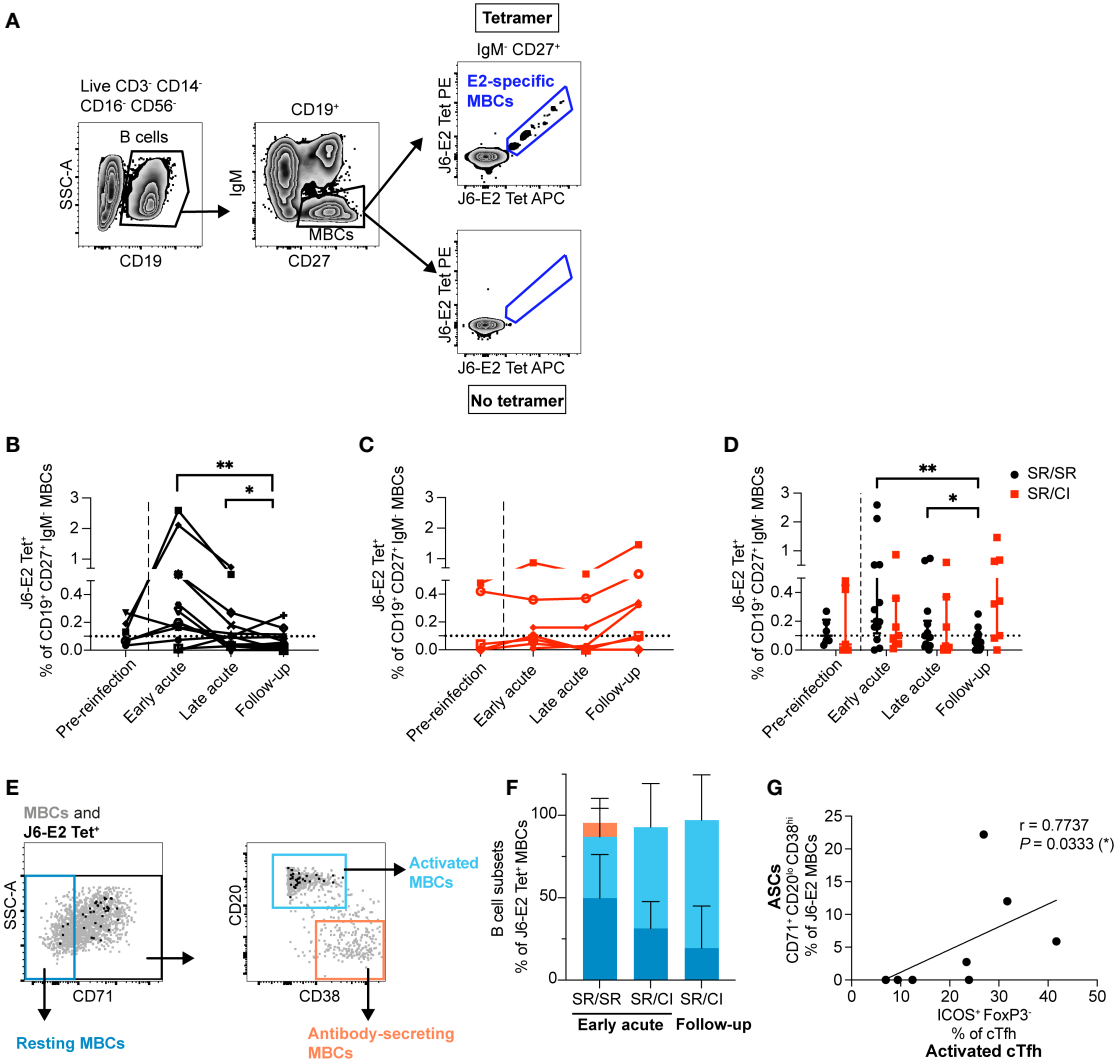
E2-specific memory B cells (MBCs) expand early and differentiate into ASCs that correlate with activated cTfh in resolvers during reinfection. **(A)** Representative gating strategy showing class-switched E2-specific MBCs using dually labeled (PE and APC) tetramers (CD3^−^CD14^−^CD16^−^CD56^−^CD19^+^CD27^+^IgM^–^J6-E2-Tet^+^). **(B, C)** Longitudinal frequencies of J6-E2 Tet^+^ MBC in SR/SR [**(B)** *n* = 14, black] and SR/CI [**(C)**, *n* = 8, red] during reinfection; dotted lines indicate the threshold for the detection of E2-specific MBCs in healthy individuals. The dashed lines delineate the reinfection episode. Two-way repeated measure ANOVA with Tukey’s *post-hoc* test. *P < 0.05; **P < 0.01. **(D)** Combined data from **(B, C)**, presenting the median with interquartile range for each group. **(E)** Representative gating strategy showing resting (CD71^–^), activated (CD71^+^CD20^hi^CD38^int-lo^), and antibody-secreting (CD71^+^CD20^lo^CD38^hi^) among the total MBC population (gray dots) and HCV E2-specific MBCs (black dots). **(F)** Phenotypic characterization of J6-E2 Tet^+^ MBC as resting (blue), activated (cyan), and antibody-secreting (orange) states; also shown are SR/SR at the early acute (*n* = 5) and SR/CI (n = 4) at the early acute and follow-up time points. **(G)** Scatter plot for Spearman’s rank correlation coefficient at the early acute time point between antibody-secreting cells (ASCs; CD71^+^CD20^lo^CD38^hi^) and activated cTfh (CD4^+^CXCR5^+^PD-1^+^ICOS^+^FoxP3^−^) from SR/SR, line of best fit, the correlation coefficient (*r*-value), and the *P*-value are shown (*P < 0.05).

Given the early expansion of activated HCV-specific cTfh observed in the SR/SR group and their essential role in helping B cells, we investigated the longitudinal dynamics of E2-specific, class-switched MBCs in both groups using HCV glycoprotein E2 tetramers (26) as previously described (17). E2-specific MBCs were identified as CD3^−^CD14^−^CD16^−^CD56^−^CD19^+^IgM^−^CD27^+^Tetramer APC^+^ and PE^+^ (**Figure 3A**). We observed early, transient, and significant expansion of E2-specific MBCs in SR/SR subjects as compared to follow-up time points (*P* = 0.0075) (**Figure 3B**), and the frequency of E2-specific MBCs remained significantly higher than the follow-up time points during the late acute stage of reinfection. During early acute reinfection, 10 out of 14 SR/SR exhibited elevated levels of E2-specific MBCs above the detection threshold. In contrast, expansion of E2-specific MBCs in SR/CI was delayed until the follow-up time points and did not reach the level of significance, with only three subjects surpassing the threshold of detection at early acute reinfection, two of which already had detectable levels at the pre-reinfection time point (**Figure 3C**). Thus, SR/SR exhibited higher and earlier expansion of E2-specific MBCs compared to the SR/CI subjects (**Figure 3D**).

We phenotyped E2-specific MBCs to distinguish between MBCs with a resting phenotype (CD71^−^), an activated phenotype (CD71^+^CD20^hi^CD38^int-lo^), and antibody-secreting cells (ASCs; CD71^+^CD20^−^CD38^hi^) (36) (**Figure 3E**). At early acute reinfection, E2-specific MBCs in both groups exhibited both an activated or resting phenotype (activated median: SR/SR = 38.2%, SR/CI = 61.6%; resting median: SR/SR = 52.9%, SR/CI = 31.3%). At the peak of E2-specific MBC expansion in SR/CI at follow-up, the cells were predominantly of the activated phenotype (median = 87%) (**Figure 3F**). Importantly, during early acute reinfection, SR/SR but not SR/CI showed a subset of ASCs, with a median of 5.9% (**Figure 3F**). The four SR/SR subjects (SR/SR-6, SR/SR-9, SR/SR-11, and SR/SR-14) who exhibited the highest expansion of activated cTfh were those who also had detectable ASCs. The frequency of ASCs correlated with the frequency of activated cTfh (**Figure 3G**). These results suggest that earlier expansion of activated cTfh1 in SR/SR may enhance the expansion of activated HCV-specific MBCs differentiated into ASCs.

### Early antibody titers in resolvers correlate with the frequency of E2-specific MBCs

**Figure 4:**
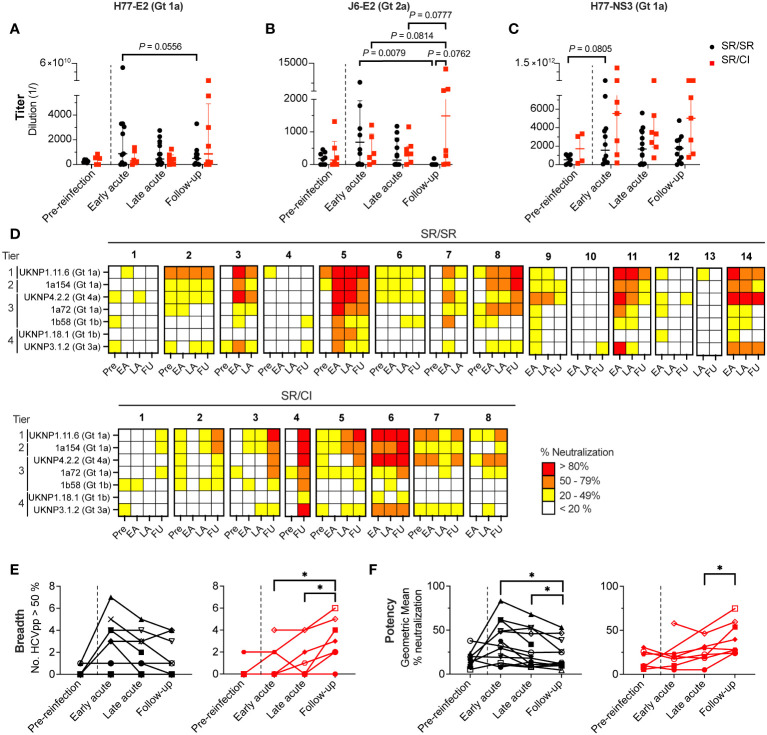
Broad and potent neutralization occurs earlier in resolvers compared to chronics. **(A–C)** Longitudinal titers of anti-HCV antibodies in the plasma of SR/SR (black) and SR/CI (red) directed against H77-E2 (Gt 1a) **(A)**, J6-E2 (Gt 2a) **(B)**, and H77-NS3 (Gt 1a) **(C)** proteins and measured by ELISA. Antigens are indicated on top of the graphs. Each symbol represents a single subject. Two-way repeated measure ANOVA with Tukey’s *post-hoc* test. **(D)** Heatmap representing the neutralizing activity of longitudinal plasma (1:50 dilution) samples of SR/SR (*n* = 14, top panels) and SR/CI (*n* = 8, bottom panels). Each sample was tested against seven HCVpp belonging to four tiers indicated on the *y*-axis. The percentage of neutralization is represented by a color. Key: white, <20%; yellow, 20%–49%; orange, 50%–79%; red, >80%. Pre, pre-reinfection; EA, early acute; LA, late acute; FU, follow-up. **(E, F)** Longitudinal neutralization breadth (**E**, number of HCVpp neutralized > 50%) and potency (**F**, geometric mean of the % of neutralization) of SR/SR (n = 14, black) and SR/CI (n = 8, red). Each symbol represents a single subject. The dashed lines delineate the reinfection episode. Two-way repeated measure ANOVA with Tukey’s *post-hoc* test. *P < 0.05.

To assess whether expansion of HCV-specific B cells is associated with enhanced production of anti-HCV antibodies, we evaluated the levels of IgG in plasma targeting HCV proteins by ELISA. We performed four-fold serial plasma dilutions to determine the antibody titer at 50% binding, defined as the dilution showing 50% binding to H77-E2 Gt 1a (**Supplementary Figure 2A**), J6-E2 Gt 2a (**Supplementary Figure 2B**), and H77-NS3 Gt 1a as a control (**Supplementary Figure 2C**). At early acute reinfection, SR/SR showed higher titers of IgG than SR/CI against H77-E2 and J6-E2 proteins, 3-fold and 2.5-fold, respectively (**Figures 4A, B**). However, these differences were not significant. Titers of anti-E2 Gt 2a in SR/SR decreased significantly (*P* = 0.0079) from early acute to follow-up time points (**Figure 4B**). In contrast, SR/CI had higher antibody titers against the H77-NS3 protein compared to SR/SR at all times during reinfection (**Figure 4C**). Overall, SR/CI reached the highest antibody titers at follow-up when chronic HCV infection was already established. Together, these results indicate that SR/SR develops earlier and higher antibody titers against E2 protein, the target for NAbs that may contribute to viral clearance.

Based on the antibody titer results (**Figures 4A–C**), we selected the OD450–570 values from 1:1,000 plasma dilution for all subsequent analyses. Longitudinal analysis of plasma antibody responses at this dilution against H77-E2 Gt 1a, J6-E2 Gt 2a, and H77-NS3 Gt 1a in SR/SR (**Supplementary Figure 3A**) and SR/CI (**Supplementary Figure 3B**) was not significantly different at early acute reinfection (**Supplementary Figure 3C**). However, the levels of anti-E2 antibodies at the early acute time point significantly and positively correlated with the frequency of E2-specific MBCs from SR/SR (H77-E2: *r* = 0.8407, *P* = 0.0006 and J6-E2: *r* = 0.7912, *P* = 0.002) (**Supplementary Figure 4A**) and SR/CI (H77-E2: *r* = 0.8571, *P* = 0.0238 and J6-E2: *r* = 1.000, *P* = 0.0004) (**Supplementary Figure 4B**). These results suggest that the recall response of E2-specific MBCs leads to increased antibody titers, potentially through the observed rapid differentiation into the ASC population (**Figure 3F**).

### Broad and potent neutralization occurs earlier in resolvers compared to chronics

Next, we investigated whether the high titers of HCV-specific antibodies were reflected in the plasma neutralization activity. We performed neutralization assays with seven HCVpp using a standardized panel (28, 37) harboring envelope glycoproteins E1E2 from HCV Gt 1a, 1b, 3a and 4a and representing different tiers of neutralization sensitivity (28, 37). The detailed results are presented in **Supplementary Figure 5** and summarized in **Figure 4D**. Seven out of 14 SR/SR neutralized >50% UKNP1.11.6 (Gt 1a) HCVpp from tier 1 (most sensitive to neutralization) compared to only two out of eight SR/CI at early acute reinfection (**Figure 4D**). However, there was no significant difference in the percent neutralization between SR/SR and SR/CI at this time point. The highest percent neutralization against this HCVpp by SR/SR (98.31%) occurred at early acute, while in the SR/CI, the highest neutralization (96.20%) was not achieved until the follow-up time point (**Supplementary Figure 5A**). Neutralization of tier 2 (1a154-H77 Gt 1a) and tier 3 (UKNP4.2.2 Gt 4a, 1a72 Gt 1a, and 1b58 Gt 1b) HCVpp followed the same trend, where SR/SR showed a higher percentage of neutralization at early acute compared to follow-up, while SR/CI reached the highest percent of neutralization only late during reinfection at follow-up time points (**Supplementary Figures 5B–E**). Regarding tier 4, the most resistant to neutralization represented by UKNP1.18.1 Gt 1b and UKNP3.1.2 Gt 3a, only SR/SR-5 neutralized >50% of both tier 4 HCVpp at early acute (**Supplementary Figures 5F, G**). At the same time point, four SR/SR (SR/SR-3, SR/SR-5, SR/SR-11, SR/SR-14) were able to neutralize >50% of the HCVpp Gt 3a versus only one chronic (SR/CI-6) (**Supplementary Figure 5G**).

We further integrated the neutralization results to define neutralization breadth and potency. Neutralization breadth, which is the number of HCVpp neutralized >50%, peaked at early acute in the SR/SR and declined with time (**Figure 4E**). In contrast, neutralization breadth in SR/CI exhibited slower kinetics, where it increased significantly only at the follow-up time points (*P* = 0.0365 and 0.0263 compared to early and late acute, respectively). Neutralization potency, which is the geometric mean of % neutralization, demonstrated a similar trend (**Figure 4F**), where the neutralization potency in SR/SR peaked at early acute, but only increased significantly in SR/CI much later at follow-up (*P* = 0.0460 compared to late acute). The early neutralization breadth and potency correlated positively and significantly in SR/SR with the levels anti-E2 Gt 1a (**Supplementary Figure 6A**, breadth: *r* = 0.8742, *P* = 0.0002 and potency: *r* = 0.8736, *P* = 0.0002) and anti-E2 Gt 2a (**Supplementary Figure 6B**, breadth: *r* = 0.7635, *P* = 0.0037 and potency: *r* = 0.7473, *P* = 0.0046) but not in SR/CI (**Supplementary Figures 6C, D**, respectively). These findings suggest that anti-E2 antibodies generated early with high titers in resolvers mediate HCVpp neutralization with high breadth and potency, thus potentially contributing to HCV clearance upon reinfection.

### Activated cTfh1 and E2-specific MBCs correlate with neutralization breadth and potency during early acute reinfection in resolvers

**Figure 5:**
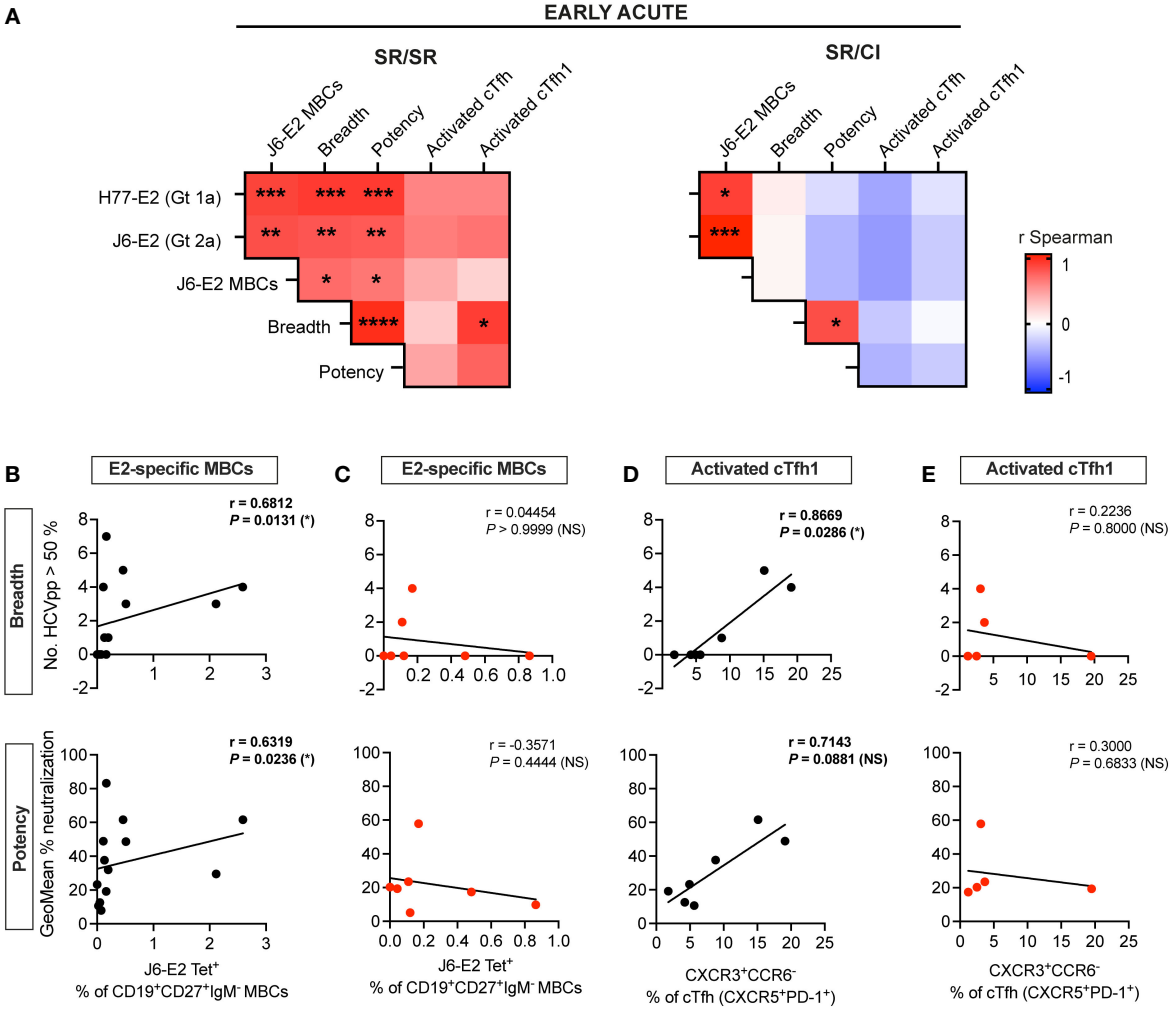
E2-specific MBCs and activated cTfh1 correlate with neutralization breadth and potency at early acute in resolvers of HCV reinfection. **(A)** Heatmaps representing the Spearman’s rank correlation coefficient (*r*) between immune responses at early acute reinfection. Data of SR/SR (left) and SR/CI (right). *P < 0.05; **P < 0.01; ***P < 0.001; ****P < 0.0001. **(B–E)** Scatter plots for Spearman’s rank correlation coefficient at early acute time points between neutralization breadth (top) or potency (bottom) with J6-E2-specific MBCs from SR/SR **(B)** and SR/CI **(C)** and with activated cTfh1 (CXCR5^+^PD-1^+^ICOS^+^FoxP3^−^CXCR3^+^CCR6^−^) from SR/SR **(D)** and SR/CI **(E)**, lines of best fit, the correlation coefficient (*r*-value), and the *P*-value are shown. NS, non-significant; *P* > 0.05; *P < 0.05.

Because of the early coordinated expansion of cTfh and E2-specific MBCs along with the elevated plasma neutralization activity in the SR/SR, we examined the correlations between these responses at early acute. Overall, we observed more significant positive associations between the different early acute immune parameters in SR/SR as compared to SR/CI as summarized in **Figure 5A** and detailed in **Supplementary Figures 7**, **8**. Notably, the frequencies of E2-specific MBCs correlated with neutralization breadth and potency in SR/SR but not SR/CI (**Figures 5B, C**). The frequencies of activated cTfh1 (ICOS^+^FoxP3^−^CXCR3^+^CCR6^−^) also correlated positively with plasma neutralizing activity in the SR/SR but not SR/CI (**Figures 5D, E**). Furthermore, at early acute reinfection, the frequencies of total activated cTfh from SR/SR but not SR/CI showed positive but non-significant correlation with anti-E2 antibodies (**Supplementary Figures 7A, B**), E2-specific MBCs (**Supplementary Figures 7C, D**), and neutralization breadth and potency (**Supplementary Figures 7E, F**). Similarly, activated cTfh1 from SR/SR but not SR/CI showed a positive non-significant correlation with anti-E2 antibodies (**Supplementary Figures 8A, B**) and E2-specific MBCs (**Supplementary Figures 8C, D**) at early acute reinfection. Altogether, these results further support that early activation of cTfh with a Th1 phenotype along with early expansion of E2-specific MBCs may drive the production of HCV-specific NAbs leading to the clearance of HCV reinfection.

## Discussion

We examined longitudinally the humoral immune response during HCV reinfections progressing to either resolution or chronicity and characterized the kinetics of cTfh and E2-specific MBCs relative to the virus-specific antibody response including IgG titers and neutralization breadth and potency. Several resolvers of HCV reinfection showed early activation of HCV-specific effector memory cTfh1 cells that are critical for humoral immunity (11, 14, 38). cTfh expansion in these was kinetically coordinated with the early expansion of activated E2-specific MBCs and ASCs. More importantly, this concerted activity was associated with high antibody titers against HCV glycoprotein E2 (Gt 1a and 2a) and increased neutralization breadth and potency of HCVpp spanning four tiers of neutralization sensitivity.

Resolvers and chronics had comparable HCV-specific T-cell responses at early acute reinfection, but resolvers showed earlier expansion and higher frequencies of activated memory cTfh, underscoring their role in the clearance of HCV reinfection. Activated cTfh became skewed toward a cTfh1 phenotype following reinfection. Indeed, the frequency of cTfh1 correlates with specific antibody responses to HCV and HIV (14, 38–41), and they represent a prominent subset of circulating HCV-specific CD4 T cells following direct-acting antiviral treatment (42, 43) and are associated with early expansion of E2-specific MBCs in resolvers of primary HCV infection (11). Upon stimulation with HCV antigens, these recall cTfh cells upregulated CD40L and OX40 that are crucial for cognate Tfh–B-cell interaction, plasma cell generation, and production of virus-specific antibodies (44, 45). Tfh help may also be mediated by IL-21 (1, 46, 47), and future studies should examine whether HCV-specific cTfh from resolvers have superior IL-21 production and/or provide better B-cell help for antibody production.

Upon reinfection or booster vaccination, virus-specific MBCs can differentiate into ASCs or re-enter the germinal center to undergo further affinity maturation with the help of Tfh cells (1). In our study, we detected the early expansion of E2-specific MBCs that differentiated into ASCs in resolvers. Notably, resolvers with detectable ASCs also showed the highest frequencies of activated cTfh, suggesting coordinated expansion of both populations. In contrast, chronics displayed delayed kinetics of E2-specific MBC expansion with a dominant activated phenotype, but no ASCs were detected. Compared to primary HCV infection (11), expansion of E2-specific MBCs upon reinfection occurred earlier and at nearly six-fold higher frequencies (up to 2.5% in reinfection versus 0.4% in primary infection). These findings suggest rapid reactivation of HCV-specific MBCs possibly due to efficient HCV-specific memory T-cell help generated following clearance of primary infection. Indeed, the upregulation of transcriptomic plasma cell signatures was associated with the clearance of HCV secondary infection (17). Interestingly, frequencies of E2-specific MBCs correlated with the levels of anti-E2 antibodies in resolvers and chronics at early acute reinfection as described during primary infection (48). Thus, E2-specific MBC expansion led to higher titers of anti-E2 antibodies in resolvers compared to chronics at early acute reinfection. It is possible that similar to reports during chronic lymphocytic choriomeningitis virus (LCMV) infection, the production of type I IFN by CD8 T cells or monocytes may have driven the differentiation of B cells into short-lived ASCs with a limited early antibody response (49–51). Plasma neutralization activity was also boosted in resolvers upon reinfection, as reported by previous studies (16, 20, 21, 52), and is probably mediated by the higher titers of anti-E2. Indeed, our observations are in line with a recently reported mechanism in which early NAbs exert selection pressure that leads to E2 substitutions followed by loss of viral fitness contributing to clearance of HCV (15, 53). Virus sequencing may resolve some of these questions; however, the low-level viremia and rapid clearance upon reinfection remain a major obstacle to this approach. Overall, resolvers showed earlier and higher plasma neutralization breadth and potency that declined with time. This early plasma neutralization activity is strongly correlated with frequencies of E2-specific MBC and cTfh1 cells in resolvers, which has been previously described for other viral infections or vaccines (38, 54–56).

Both T- and B-cell responses contribute to the clearance of HCV reinfection, but very few studies have examined both simultaneously. Resolution of HCV reinfection was associated with expansion of virus-specific memory CD4 and CD8 T cells (17, 18, 24, 57) and was dependent upon cross-recognition of the reinfection virus sequence by pre-existing memory T cells (24). When both responses were examined, different patterns and kinetics of T- and B-cell responses were detected in resolving reinfections (18). In the present study, we also observed heterogeneity within the cTfh, B cells, and NAb responses in resolvers. Similarly, two recent studies reported variable patterns of NAbs during HCV reinfection (20, 21). Altogether, these data suggest different paths to HCV clearance where each arm of the adaptive immune response may contribute to various degrees. Although we could not assess viral sequence in the present study, it is tempting to speculate that in the case of viral escape from the CD8 T cells, help from Tfh to support the production of NAbs and functional MBCs (20) becomes more crucial in the control of virus replication.

There are a few limitations to this study. First, because of the limited availability of the samples, we focused primarily on the analysis of AIM^+^ cells at the early acute time points where resolvers showed higher frequencies of activated cTfh and E2-specific MBCs. Second, because we used J6-E2 (Gt 2a) tetramers, we may have missed MBCs recognizing non-conserved epitopes in the E2 protein. The use of the full-length envelope protein (E1E2) would be of interest in future studies to enhance the detection of HCV-specific MBCs. Third, we did not define the epitopes and structural characteristics of NAbs isolated from resolvers versus chronics that may influence neutralization efficacy. However, data from previous studies identified potent NAbs targeting similar epitopes in resolvers and chronics (53, 58–60). So far, data suggest that it is the timing of the appearance of NAbs rather than their quality that is the main determinant of spontaneous clearance of HCV infection and reinfection.

Our results suggest an important role of Tfh cells during recall responses to human HCV infection, and cTfh1 cells provide help to MBCs for the generation of potent NAbs that contribute to the rapid clearance of reinfection upon re-exposure. Future studies examining the molecular mechanisms implicated in preferential expansion of these subsets in resolvers should provide potential targets for enhancing the immune response to next-generation vaccines against HCV and other viruses.

## Data availability statement

The raw data supporting the conclusions of this article will be made available by the authors, without undue reservation.

## Ethics statement

The studies involving humans were approved by the Research Ethics Committee of the Centre de Recherche du Centre Hospitalier de l’Université de Montréal (CRCHUM) (Approval number: SL 05.014). The studies were conducted in accordance with the local legislation and institutional requirements. The participants provided their written informed consent to participate in this study.

## Author contributions

ME: Conceptualization, Data curation, Formal analysis, Investigation, Methodology, Validation, Visualization, Writing – original draft, Writing – review & editing. EG-E: Conceptualization, Data curation, Formal analysis, Investigation, Methodology, Visualization, Writing – original draft, Writing – review & editing. NB: Data curation, Investigation, Methodology, Project administration, Validation, Visualization, Writing – review & editing. NA: Data curation, Formal analysis, Investigation, Methodology, Writing – review & editing. NF: Data curation, Investigation, Methodology, Writing – review & editing. SM: Data curation, Formal analysis, Investigation, Writing – review & editing. AF-B: Data curation, Investigation, Writing – review & editing. PS: Data curation, Investigation, Writing – review & editing. JG: Methodology, Writing – review & editing. MA-H: Data curation, Formal analysis, Investigation, Methodology, Writing – review & editing. JB: Writing – review & editing, Conceptualization, Funding acquisition, Investigation, Resources. AG: Conceptualization, Funding acquisition, Supervision, Validation, Writing – review & editing, Resources. NS: Conceptualization, Funding acquisition, Project administration, Supervision, Validation, Writing – original draft, Writing – review & editing.
