# Supplementary material for: Coordinated expansion of memory T follicular helper and B cells mediates spontaneous clearance of HCV reinfection
Source: Front Immunol. 2024 Jun 10;15:1403769. doi: 10.3389/fimmu.2024.1403769 (PMC11211980; doi:10.3389/fimmu.2024.1403769)
Supplement: Supplementary file 1 [file DataSheet_1.pdf]

Supplementary Material

Coordinated expansion of memory T follicular helper and B cells mediate spontaneous clearance of HCV reinfection

Mohamed Eisa[1,†], Elsa Gomez-Escobar[1,2,†], Nathalie Bédard[1], Nourtan F. Abdeltawab[1,3], Nicol Flores[1,2], Sabrina Mazouz[1,2], Alizée Fieffé-Bédard[1], Patrick Sakayan[1], John Gridley[4], Mohamed S. Abdel-Hakeem[1,2,3,7], Julie Bruneau[1,5], Arash Grakoui[4], Naglaa H. Shoukry[1,6,*]

[1]Centre de Recherche du Centre Hospitalier de l'Université de Montréal (CRCHUM), Montréal, QC, Canada
[2]Département de microbiologie, virologie et immunologie, Université de Montréal, Montréal, QC, Canada
[3]Department of Microbiology and Immunology, Faculty of Pharmacy, Cairo University, Cairo, Egypt
[4]Department of Medicine, Emory University, Atlanta, USA
[5]Département de médecine familiale et département d'urgence, Université de Montréal, Montréal, QC, Canada
[6]Département de médecine, Université de Montréal, Montréal, QC, Canada.
[7]Current address: Department of Pathology and Laboratory Medicine, Emory University, Atlanta, USA

†These authors share first authorship

***Correspondance:**
Dr. Naglaa Shoukry
Centre de Recherche du CHUM (CRCHUM),
Tour Viger, Local R09.414,
900 rue St-Denis,
Montréal, QC H2X 0A9 CANADA
E-mail: naglaa.shoukry@umontreal.ca

SUPPLEMENTARY TABLES

Supplemental Table 1. Detailed clinical data of reinfection subjects.

Sheet 1. Resolvers

Sheet 2. Chronics

Supplemental Table 2. Flow cytometry antibodies.

**Panel #1 Identification of E2-specific memory B cells (MBCs)**				
**Antigen**	**Fluorophore**	**Clone**	**Catalog#**	**Provider**
CD3	V500	UCHT1	561416	BD Biosciences
CD14	V500	M5E2	561391	BD Biosciences
CD16	V500	3G8	561394	BD Biosciences
CD19	BUV496	SJ25C1	612938	BD Biosciences
CD20	BV785	2H7	302356	BioLegend
CD21	BUV737	B-ly4	612788	BD Biosciences
CD27	R718	M-T271	567678	BD Biosciences
CD38	BUV395	563811	HB7	BD Biosciences
CD56	BV510	NCAM16.2	563041	BD Biosciences
CD71	PerCP-Cy5.5	CY1G4	334114	BioLegend
IgM	BB515	G20-127	564622	BD Biosciences
**Panel #2 cTfh phenotyping**				
CD3	BUV496	UCHT1	612940	BD Biosciences
CD4	BV605	RPA-T4	562658	BD Biosciences
CD8	BUV395	RPA-T8	563795	BD Biosciences
CD45RA	AF700	HI100	560673	BD Biosciences
CD183 (CXCR3)	APC	1C6/CXCR3	550967	BD Biosciences
CD185 (CXCR5)	PerCP-Cy5.5	RF8B2	562781	BD Biosciences
CD196 (CCR6)	BV786	11A9	563704	BD Biosciences
CD278 (ICOS)	PE	DX29	557802	BD Biosciences
CD279 (PD1)	BV421	EH12.1	562516	BD Biosciences
FoxP3	PE-Cy7	PCH101	25-4776-42	eBioscience
**Panel #3 AIM assay**				
CD3	BUV496	UCHT1	612940	BD Biosciences
CD4	BUV805	RPA-T4	742000	BD Biosciences
CD8	APC-H7	SK1	560179	BD Biosciences
CD14	V500	M5E2	561391	BD Biosciences
CD19	V500	HIB19	561121	BD Biosciences
CD45RA	FITC	HI100	555488	BD Biosciences
CD69	APC	FN50	560711	BD Biosciences
CD134 (OX40)	PE-CF594	ACT35	563662	BD Biosciences
CD137 (4-1BB)	R718	4B4-1	567086	BD Biosciences

CD154 (CD40L)	BUV563	24-31	752854	BD Biosciences
CD183 (CXCR3)	PE-Cy7	1C6/CXCR3	560831	BD Biosciences
CD185 (CXCR5)	BV750	RF8B2	747111	BD Biosciences
CD194 (CCR4)	BUV615	1G1	613000	BD Biosciences
CD196 (CCR6)	BUV737	11A9	612780	BD Biosciences
CD197 (CCR7)	BV650	3D12	563407	BD Biosciences
PD-1 (CD279)	BV711	EH12.1	564017	BD Biosciences
CD278 (ICOS)	BUV661	DX29	741664	BD Biosciences

SUPPLEMENTARY FIGURES

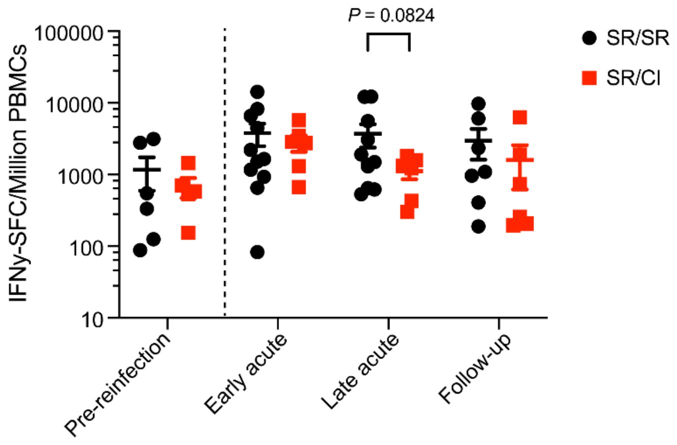

Supplemental Figure 1. Sustained HCV-specific T-cell responses in resolvers compared to chronics during reinfection.

Frequencies of IFN-γ spot-forming cell (SFC) per million PBMCs from SR/SR (n=12, black) and SR/CI (n=8, red). Two-way repeated measure ANOVA with Tukey's post-hoc test.

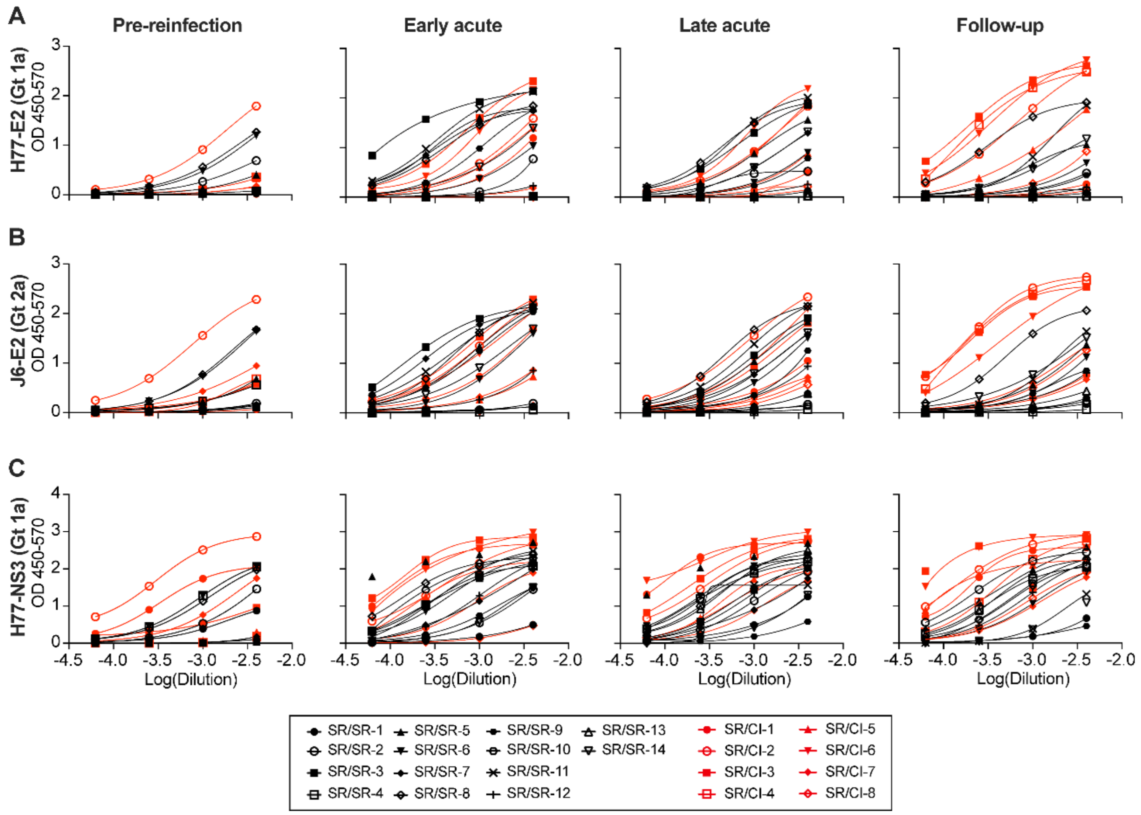

Supplemental Figure 2. Serial dilutions of plasma show differential binding to HCV E2 and NS3 in resolvers and chronics during reinfection.

Longitudinal anti-H77-E2 (Gt 1a) (**A**), anti-J6-E2 (Gt 2a) (**B**) and anti-H77-NS3 (Gt 1a) (**C**) IgG responses in the plasma (four-fold dilutions) of SR/SR (n=14, in black) and SR/CI (n=8, in red) measured by ELISA, and represented as OD450–570. Antigens are indicated to the far left of each row, and time points at the top of each column. Each symbol represents a single subject as indicated at the bottom legend.

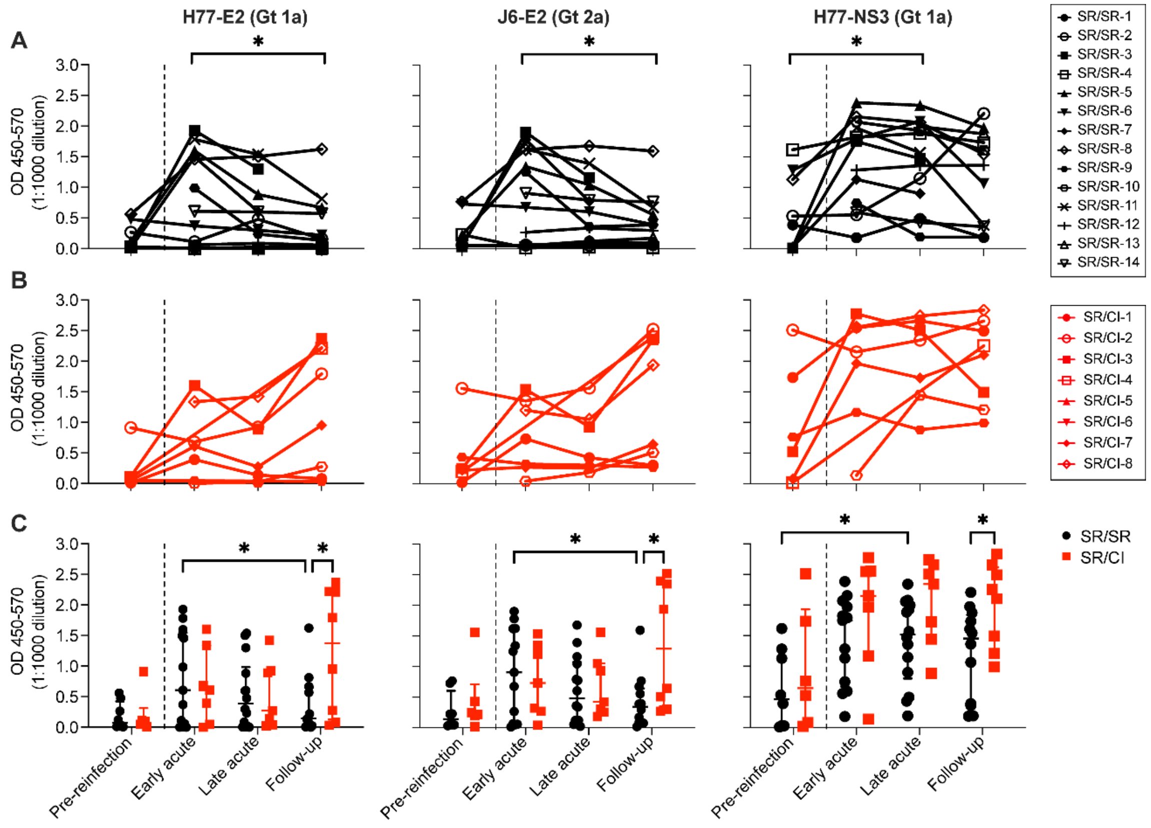

Supplemental Figure 3. Higher levels of plasma anti-HCV-E2 antibodies at early acute reinfection in resolvers. Longitudinal anti-H77-E2 (Gt 1a), anti-J6-E2 (Gt 2a) and anti-H77-NS3 (Gt 1a) IgG responses in the plasma (at 1:1000 dilution) of SR/SR (**A**) (n=14, in black) and SR/CI (**B**) (n=8, in red) measured by ELISA. (**C**) Combined data from A and B, presenting the median with interquartile range for each group. Two-way repeated measure ANOVA with Tukey's post hoc test. $*P < 0.05$.

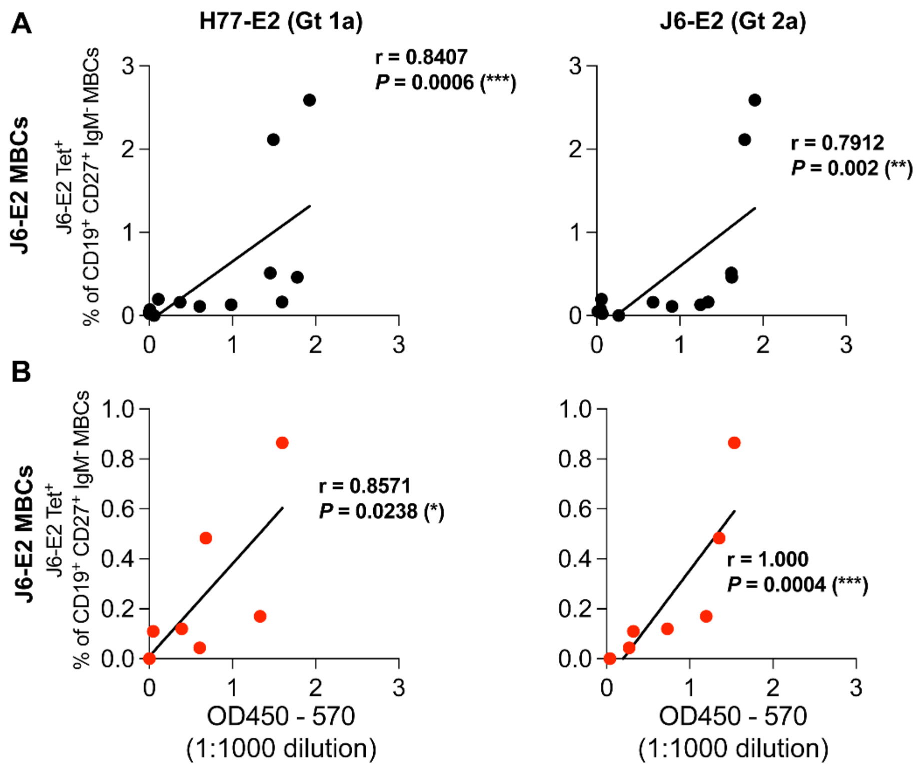

Supplemental Figure 4. Frequency of E2-specific MBCs correlates with plasma anti-HCV-E2 antibody levels at early acute reinfection.

Scatter plots for Spearman's rank correlation coefficient between E2-specific MBCs and anti-H77-E2 (Gt 1a) (left) or anti-J6-E2 (Gt 2a) (right) in SR/SR (**A**) and SR/CI (**B**) at early acute time points of reinfection. lines of best fit, the correlation coefficients (r-values) and 95% confidence interval are shown. *$P < 0.05$, **$P < 0.01$; ***$P < 0.001$.

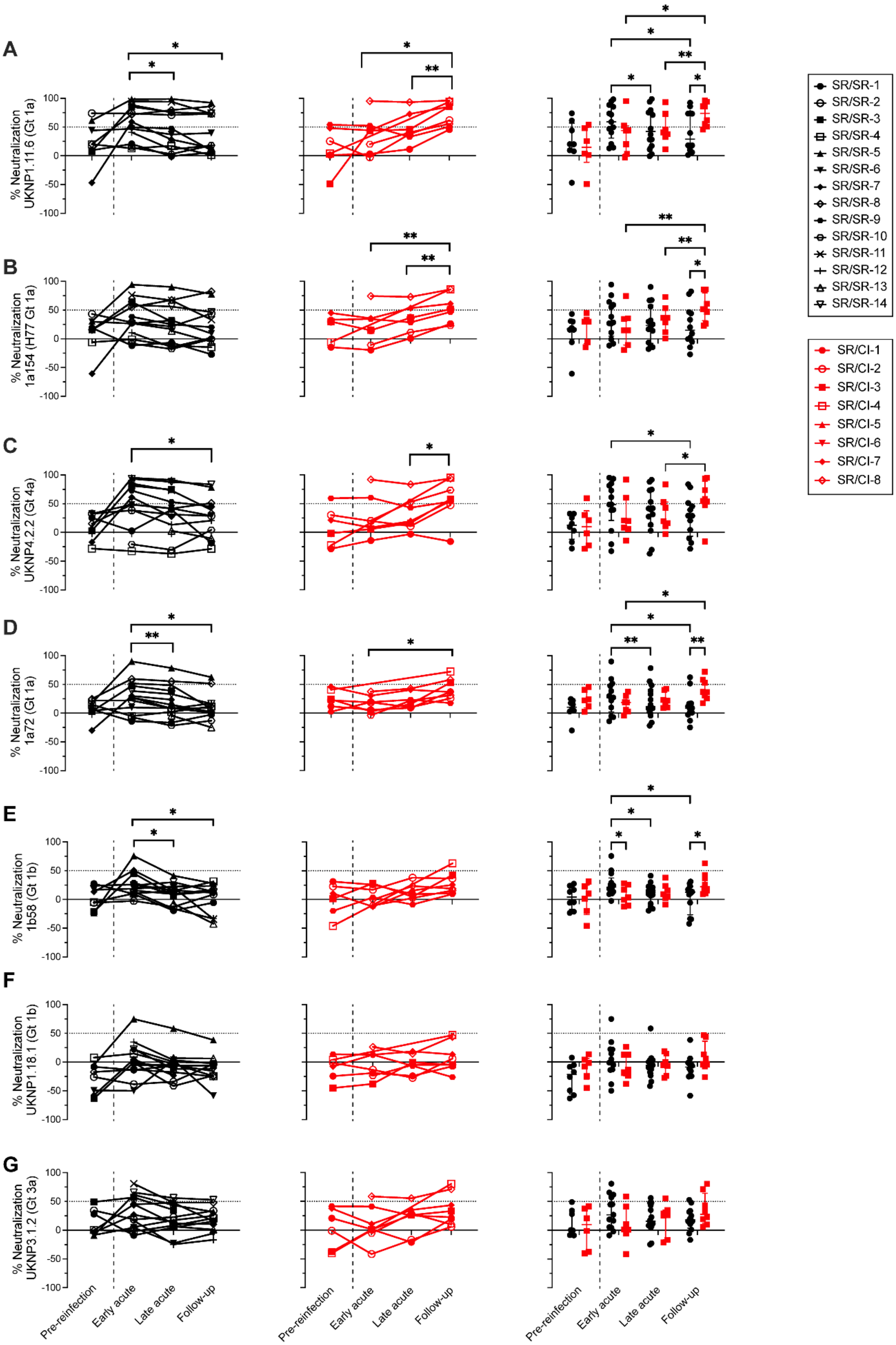
A
B
C
D
E
F
G
% Neutralization UKNP1.11.6 (Gt 1a)
% Neutralization 1a154 (H77 Gt 1a)
% Neutralization UKNP4.2.2 (Gt 4a)
% Neutralization 1a72 (Gt 1a)
% Neutralization 1b58 (Gt 1b)
% Neutralization UKNP1.18.1 (Gt 1b)
% Neutralization UKNP3.1.2 (Gt 3a)
100
50
0
-50
-100
Pre-reinfection
Early acute
Late acute
Follow-up
SR/SR-1
SR/SR-2
SR/SR-3
SR/SR-4
SR/SR-5
SR/SR-6
SR/SR-7
SR/SR-8
SR/SR-9
SR/SR-10
SR/SR-11
SR/SR-12
SR/SR-13
SR/SR-14
SR/CI-1
SR/CI-2
SR/CI-3
SR/CI-4
SR/CI-5
SR/CI-6
SR/CI-7
SR/CI-8

Supplemental Figure 5. Antibodies from resolvers show higher neutralization activity at early acute reinfection as compared to chronics.

Longitudinal analysis of percentage neutralization by the plasma of SR/SR (black) and SR/CI (red) against 7 HCV pseudoparticles from Tier 1: UKNP1.11.6 (Gt 1a) (**A**), Tier 2: 1a154 (Gt 1a) (**B**), Tier 3: UKNP4.2.2 (Gt 4a), 1a72 (Gt 1a) and 1b58 (Gt 1b) (**C**, **D** and **E** respectively), and Tier 4: UKNP1.18.1 (Gt 1b) and UKNP3.1.2 (Gt 3a) (**F** and **G**). The doted lines indicate 50% neutralization, and the dashed lines delineate the reinfection episode. Two-way repeated measure ANOVA with Tukey's post hoc test. $^{*}P < 0.05$, $^{**}P < 0.01$.

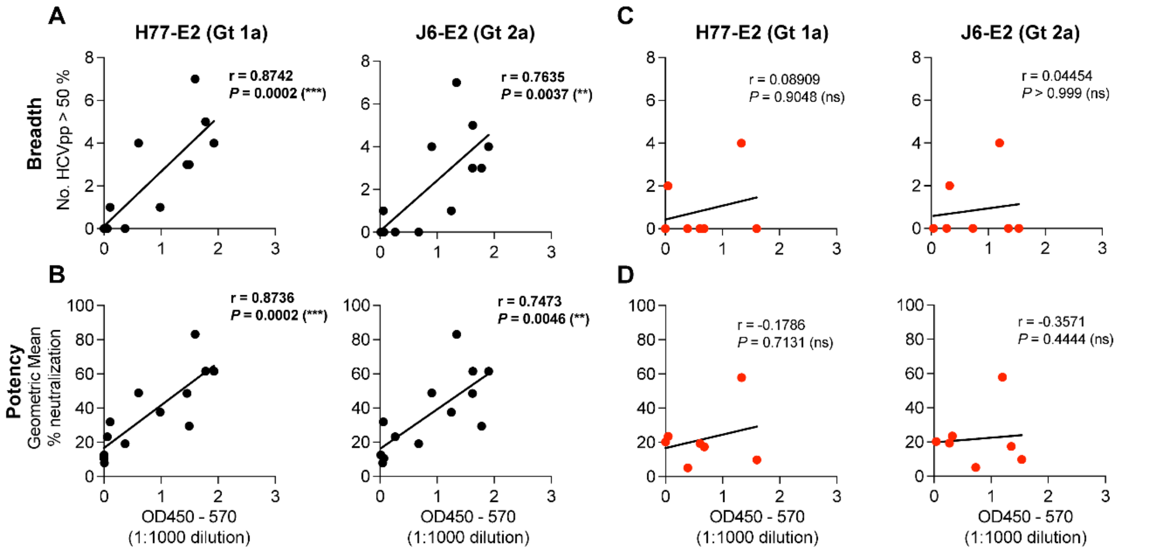

Supplemental Figure 6. Neutralization breadth and potency correlate with anti-HCV-E2 antibodies in resolvers but not chronics at early acute reinfection.
Scatter plots for Spearman's rank correlation coefficient of neutralization breadth (top) or potency (bottom) with anti-H77-E2 (Gt 1a) or anti-J6-E2 (Gt 2a) in SR/SR (**A** and **B**, respectively) and SR/CI (**C** and **D**, respectively) at early acute time points of reinfection. lines of best fit, the correlation coefficients (r-values) and 95% confidence interval are shown. NS (non-significant), $**P < 0.01$; $***P < 0.001$.

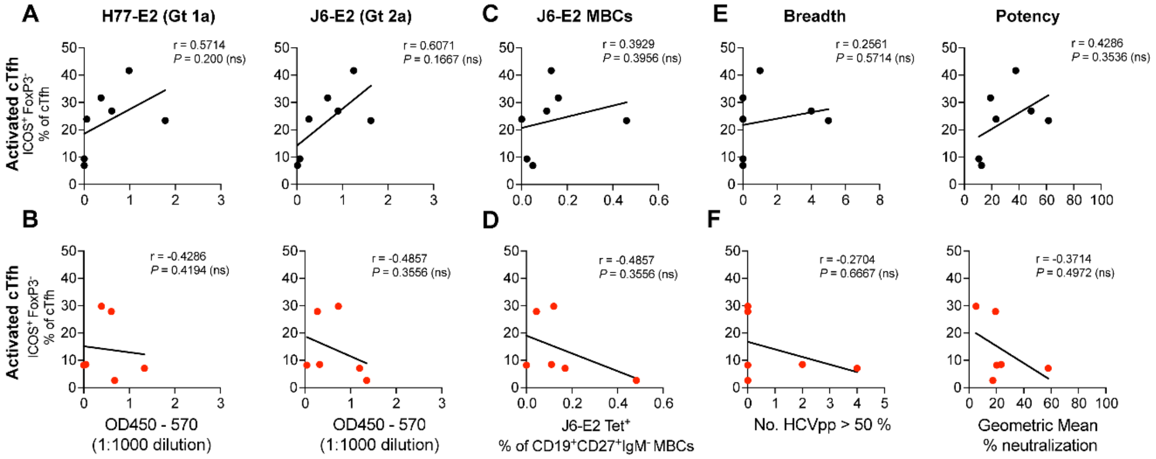

Supplemental Figure 7. Frequency of total activated cTfh cells correlates with plasma levels of anti-HCV-E2 antibodies, frequency of E2-specific MBCs, and neutralization breadth and potency in resolvers but not chronics at early acute reinfection.

(**A** - **F**) Scatter plots for Spearman's rank correlation coefficient for total activated cTfh with anti-E2 antibodies in SR/SR (**A**) and SR/CI (**B**), or with E2-specific MBCs in SR/SR (**C**) and SR/CI (**D**) or with neutralization breadth and potency in SR/SR (**E**) and SR/CI (**F**) at early acute time points of reinfection. lines of best fit, the correlation coefficients (r-values) and 95% confidence interval are shown. NS (non-significant).

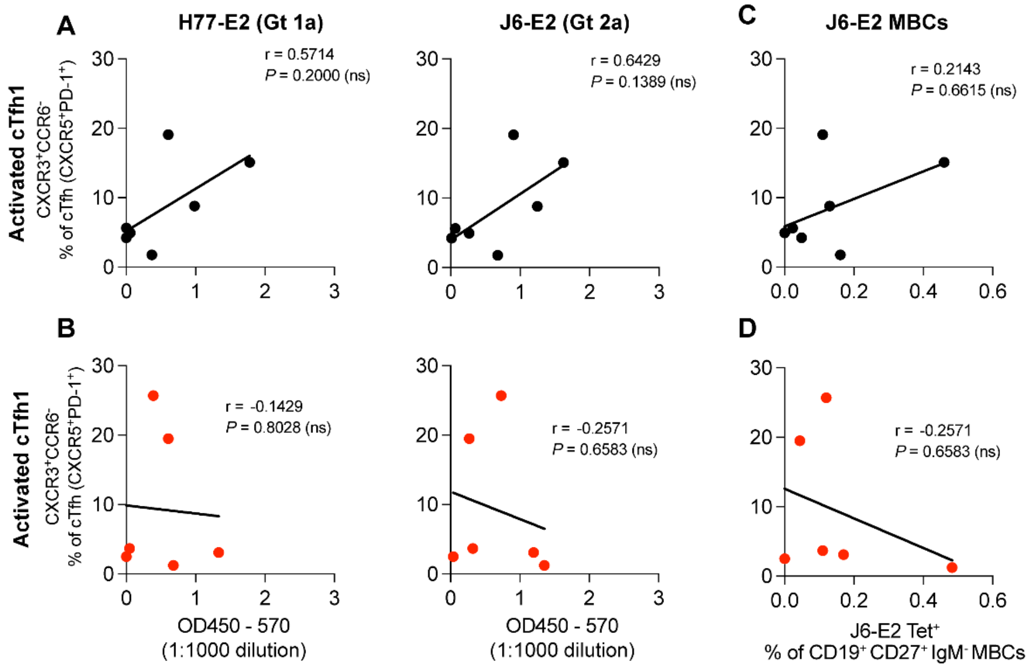

Supplemental Figure 8. Frequency of activated cTfh1 cells correlates with plasma levels of anti-HCV-E2 antibodies and frequency of E2-specific MBCs in resolvers but not chronics at early acute reinfection.

(**A - D**) Scatter plots for Spearman's rank correlation coefficient for activated cTfh1 ($CXCR5^+$ $PD\text{-}1^+ICOS^+$ $FoxP3^-CXCR3^+CCR6^-$) with anti-E2 antibodies in SR/SR (**A**) and SR/CI (**B**) or with E2-specific MBCs in SR/SR (**C**) and SR/CI (**D**). lines of best fit, the correlation coefficients (r-values) and 95% confidence interval are shown. NS (non-significant).
